# Photobiomodulation therapy increases neural stem cell pool in aged 3xTg-AD mice

**DOI:** 10.1371/journal.pone.0321668

**Published:** 2025-04-22

**Authors:** Kevin J. Johnson, Kathia Johnson, Auston Grant, Giulio Taglialatela, Maria-Adelaide Micci

**Affiliations:** 1 Department of Anesthesiology, University of Texas Medical Branch, Galveston, Texas, United States of America; 2 Department of Neurobiology, Neuroscience Graduate Program, University of Texas Medical Branch, Galveston, Texas, United States of America; 3 The Mitchell Center for Neurodegenerative Disorders, Department of Neurology, University of Texas Medical Branch, Galveston, Texas, United States of America; University of Rijeka Faculty of Medicine: Sveuciliste u Rijeci Medicinski fakultet, CROATIA

## Abstract

Presently approved Alzheimer’s Disease (AD) therapeutics are designed for targeted removal of the AD-related toxic protein aggregate amyloid-β (Aβ) and have only shown moderate efficacy at slowing disease progression. Reversal of cognitive decline requires both removal of toxic aggregates and repair of the cellular systems damaged by decades of exposure to these aggregates. Adult hippocampal neurogenesis (AHN) is one such system that is known to be affected early and severely in the development of AD. Moreover, preserved AHN is associated with cognitive resilience to AD neuropathology. Therefore, targeted therapies to improve or enhance neurogenesis should be considered in addition to the removal of toxic protein aggregates. Photobiomodulation (PBM) using 670 nm LED light has been shown to induce synaptic resilience to and removal of AD-related toxic protein aggregates. In this study, we aimed to assess the effect of PBM on a mouse model of advanced AD neuropathology. Transgenic 3xTg-AD mice (15- to 17-month old) were randomized to receive PBM or SHAM therapy for one month, followed by neuropathological assessments. Our results show that one month of PBM therapy reduces hyperphosphorylated tau burden and partially rescues AHN in aged 3xTg-AD mice as compared to SHAM-treated transgenic mice. These data support the notion that PBM has the potential to be an effective non-invasive therapy to help preserve AHN and reduce cognitive dysfunction in moderate to advanced AD.

## Introduction

Alzheimer’s Disease (AD) is the most common age-related neurodegenerative disease worldwide for which there is no resolving cure. Currently, 6.7 million Americans age 65 and older are living with AD, with incidence nearly tripling every ten years after age 65 [1,2]. In 1991, the amyloid cascade hypothesis was proposed positing that AD occurred by a sequential cascade of amyloid-β (Aβ) deposition, followed by tau neurofibrillary tangle formation and finally neuronal death [3–5]. Since that time, the vast majority of therapeutic developments have been directed towards the inhibition of amyloid-β (Aβ) and tau formation, and have been unsuccessful at reversing cognitive decline in AD patients despite reductions in pathological biomarkers [6,7]. However recent developments from long-term follow up studies of anti-Aβ immunotherapy trials have shown modest success in reducing neuropathology and slowing cognitive decline suggesting that early and continuous intervention could yield clinically significant reductions in AD symptoms [8–10]. Unfortunately by the time patients present with cognitive decline, Aβ and hyperphosphorylated tau deposition, synaptic dysfunction, hippocampal atrophy, and declining neurogenesis are suggested to have been occurring for years to decades [11–13]. Effective treatment of these patients requires reversal of these neurodegenerative pathologies in conjunction with the removal of their supposed cause. To this end, we investigated the efficacy of chronic administration of transcranial 670nm continuous wave photobiomodulation (PBM), using a commercially available device, to reverse AD neuropathology in an animal model of advanced AD with a specific focus on its effects on hippocampal neurogenesis.

Adult hippocampal neurogenesis (AHN) is the process by which multipotent stem cells in the subgranular zone (SGZ) of the hippocampal dentate gyrus (DG) differentiate and mature into functional granule neurons of the DG granule cell layer [14]. In addition to holding the potential for generation and integration of new neurons into hippocampal circuitry, neural stem cells (NSC) themselves confer neuroprotective effects against Aβ oligomers via secreted exosomes and their preservation is associated with cognitive resilience in non-demented individuals with AD neuropathology [15,16]. Therefore, targeting AHN is a viable strategy for both resilience against and potential reversal of cognitive decline in AD.

Previous studies have shown that chronic administration of 670nm PBM induces synaptic resilience to Aβ oligomers and reduces Aβ deposition in the amyloidogenic Tg2576 mouse model [17], and reduces oligomeric tau, and increases markers of autophagy at the synapses of 12-month-old 3xTg-AD mice [18]. PBM at 670nm has also been shown to significantly reduce inflammatory markers in the spinal cords of mice with induced autoimmune encephalomyelitis [19]. Other studies using varying wavelengths and modalities of PBM have shown pro-neurogenic effects in the context of acute brain injury [20,21]. To our knowledge, no studies have assessed the effects of 670nm PBM in an aged 3xTg mouse model of advanced AD.

## Methods

### Animals

To determine the effects of PBM in an advanced model of AD pathology, female 3xTg-AD (3xTg; APP_SWE_, PS1_M146V_, tau_P301L_) [22], mice between 15- and 17-months of age (mean at conclusion of treatment: 16.16 ± 0.28 months) were randomized to receive PBM or SHAM treatment.. Wild type (WT), female B6129SF1 mice were bred in house by crossing C57/Bl6J and 129S1/SvImJ (Jackson Laboratories, Bar Harbor, ME), as this is the same hybrid background strain of the 3xTg-AD mice and used as controls to confirm the AD phenotype (behavior and pathology) of the transgenic mice. All experiments performed in this study were approved and conducted in accordance with the Institutional Animal Care and Use Committee of the University of Texas Medical Branch (0910062D, 10 September 2021). Animals were housed in cages of three to five mice under a 12-hour light dark cycle with access to food and water ad libitum in the UTMB Animal Resource Center vivarium. All treatments were performed during the light cycle.

### PBM treatments

PBM treatments were performed as described previously [17]. Briefly, awake animals that had been previously habituated to handling by a single experimenter for one week prior to the start of treatment to minimize stress were manually restrained with the body of the animal covered in a reflective barrier to ensure localized treatment to the head. A 670nm WARP10 light-emitting diode (LED) device (Quantum WARP Devices, Barneveld, WI, USA), which emits 50mW/cm^2^ from a 10 cm^2^ LED array for 90 seconds for a total energy dose of 45J at a fluence of 4.5J/cm^2^, was held approximately 1 cm from the top of the head (3xTg + PBM, n=9). The area of the mouse head irradiated by the LED array is ~0.72 cm^2^, to more accurately determine the energy dose reaching the mouse scalp at each treatment considering the possibility of non-uniformity within the LED array, we used a Spectra-Physics analog power meter to measure the intensity of the light through a 0.72 cm^2^ aperture at a distance of 1 cm as 28mW resulting in an approximate fluence of 3.52J/cm^2^ delivered to the surface of the mouse scalp over the 90 second treatment. Littermate control 3xTg (3xTg Sham, n=8) and WT B6129SF1 control (WT ctrl, n=4) animals were handled in the same way and held beneath the LED device with the reflective cover for 90 seconds with the device powered off. All treatments occurred during the first four hours of the light cycle. Animals received one treatment per day, five days per week for four weeks.

### Tissue collection

On day 20 of PBM treatment, animals were sacrificed by isoflurane anesthesia followed by transcardial perfusion with heparinized phosphate-buffered saline. Brains were removed and bisected into left and right hemispheres with the latter post-fixed in 4% paraformaldehyde (PFA) for immunofluorescence analysis, and the former dissected to remove and flash freeze the hippocampus for western blot analysis.

### Immunofluorescence

PFA-fixed right brain hemispheres were sent to Neuroscience Associates (NSA, Knoxville, TN) for processing using their proprietary Multibrain® technology. Briefly, up to forty mouse hemibrains were embedded in a single gel block and sectioned at 35μm thickness in the coronal plane. Up to six sections representing different regions of the hippocampus were immunostained with the following primary antibodies: Sox2 (Abcam), doublecortin (DCX, Santa Cruz), calretinin (CR, SWANT), β-amyloid (6E10, Biolegend), phospho-tau (pTau, AT8, Invitrogen), βIII-tubulin (Abcam), Iba1 (Synaptic Systems), and CD68 (Rockland). Stained sections were imaged at 20X magnification using a Keyence BZ-X fluorescent microscope (Keyence Corporation, Osaka, Japan). Multi-tile z-stack images were stitched and Z projected onto a single plane to visualize the whole hippocampus or DG then blinded for quantitative analysis. Three to six slides were quantified per analysis. For DCX count and area analyses, single channel images for the target marker were converted to 8-bit and analyzed in ImageJ using a predetermined threshold applied to quantify the area above threshold within a given cell. Quantification and area analysis of Aβ plaques and Iba1+ microglia were conducted using Imaris image analysis software (Oxford Instruments, Concord, MA)

### DCX+ neuronal morphology

To characterize dendritic morphology of DCX+ newborn neurons, confocal images of individual DCX+ cells were taken at 60X magnification using an Olympus FV1200 laser scanning confocal microscope. Twelve slides were stained with DCX for confocal analysis. Z-stacks were uploaded into Imaris for 3D morphological analysis using the automated Filament tracer. To quantify dendritic arborization complexity, filaments were analyzed using the Sholl technique [23] with a 5µm interval between concentric spheres. Sholl intersections were averaged for each animal prior to statistical analysis to avoid confounding clustering effects.

### Plaque-associated inflammatory environment

To assess the inflammatory microenvironment surrounding extracellular Aβ deposits (plaques) in aged 3xTg-AD mice, hippocampal brain sections were stained for Aβ (6E10), the microglial marker Iba1, and the lysosomal marker CD68. A minimum of three plaques per animal were imaged at 60X magnification using an Olympus FV1200 laser scanning confocal microscope. Plaque number, size, and microglial association were consistent between treatment groups, animals in which no plaques were identified were excluded from this analysis (S1 Fig). Z-stacks were reconstructed for volumetric analysis using Imaris. Microglia indicated by Iba1+ staining were classified into three categories based on shortest distance to plaques: <0μm for plaque-associated microglia, 0–50μm, and 50–150μm for non-plaque associated microglia. Microglial morphology and CD68 content were quantified for each category.

### Western blotting

Western blot analysis was performed on whole hippocampal tissue lysed in radio-immunoprecipitation assay (RIPA) buffer (Thermo Fisher, St. Louis, MO) with protease and phosphatase inhibitors. Protein concentration was measured using a Pierce BCA assay (Thermo Fisher) and 25μg protein were loaded per sample. Electrophoretic separation was run using Bio-Rad Mini-Critereon TGX Stain-Free precast gels (Bio-RAD, Hercules, CA), and gels were activated under UV light to visualize total protein distribution. Separated proteins were transferred to PVDF membrane (Bio-RAD) and imaged again to acquire total protein concentration for normalization (S2 Fig), then blocked using EveryBlot blocking buffer (Bio-RAD) before primary antibody incubation overnight. Primary antibodies used were as follows: Tau5 (total tau; Invitrogen), AT8 (phospho-tau pSer202, pThr205; Invitrogen). Membranes were then incubated with the appropriate HRP-conjugated secondary antibody and bands visualized with SuperSignal West Pico PLUS enhanced chemiluminescence substrate (Thermo Fisher). Membranes were imaged using a ChemiDoc XRS+ System (Bio-RAD) and bands were quantified by total protein normalization using ImageLab software (Bio-RAD).

### Statistical analysis

Data were statistically analyzed using Graphpad Prism Software (Boston, MA). One-way ANOVA was used to determine statistical significance between WT control, 3xTg Sham, and 3xTg + PBM groups for all quantifications unless specified otherwise. To analyze plaque-associated inflammatory microenvironment in PBM- vs. Sham-treated 3xTg mice, between- and within-group comparisons were tested using two-way ANOVA with Tukey’s multiple comparisons test. Sholl intersection data for each animal were analyzed by two-way repeated measures ANOVA to test for statistical variance in the number of intersections between treatment groups and Sholl radii, and multiple comparisons ANOVA to compare Sholl intersections between each treatment group at each Sholl radius. All data are represented as mean ± standard deviation.

## Results

### PBM increases neural stem cell pool without altering neurogenesis in aged 3xTg-AD mice

**Fig 1:**
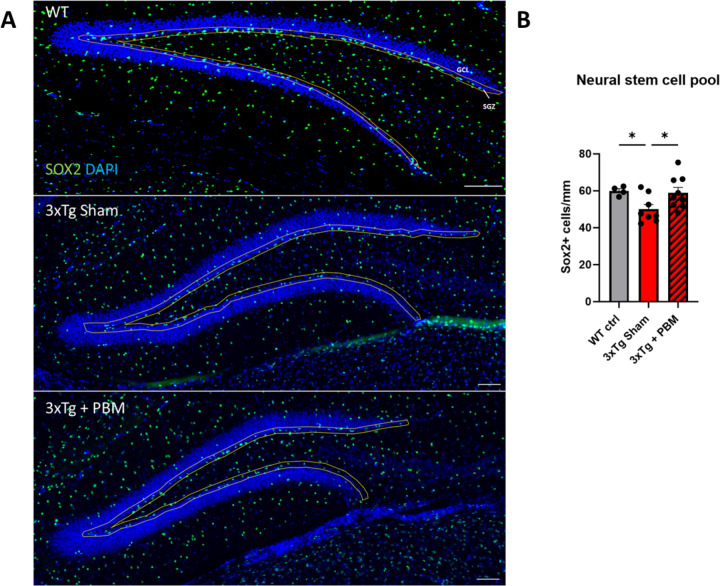
PBM therapy restores neural stem cell pool to WT levels in 3xTg mice. **A.** 20X multi-panel images were stitched together to quantify sox2+ cells in the dentate gyrus subgranular zone (outlined in yellow, scale bars = 100µm). **B**. NSC quantification was done using sox2+ cell counts normalized to SGZ length (mm). (One-way ANOVA *p<0.05).

**Fig 2:**
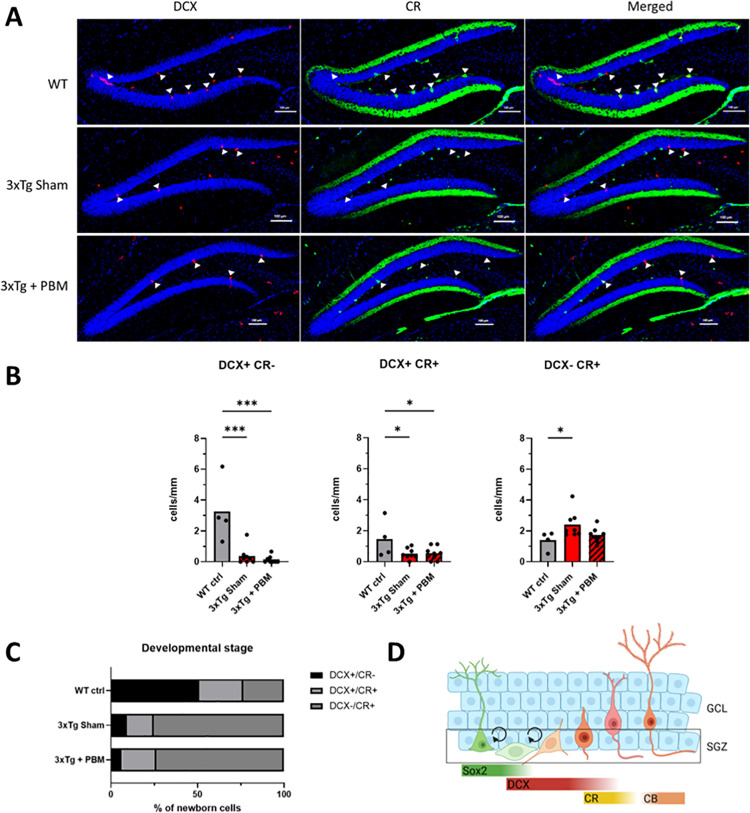
PBM does not affect the rate of neurogenesis in aged 3xTg mice. **A.** Representative images of DCX (red) and calretinin (CR, green) stained dentate gyrus sections showing DCX+ cells (arrowheads) and CR+ cells in the subgranular zone and granule cell layer (Scale bars = 100µm) **B.** Quantification of DCX and CR staining showing significant reductions in the number of newborn neurons in both 3xTg groups compared to WT with no effect of PBM. (One-way ANOVA. *p<0.05, **p<0.01, ***p<0.001). **C.** Analysis of newborn granule neurons at different stages of development shows a dramatic decrease in newly generated DCX+ neural progenitors and an overrepresentation of DCX-/CR+ immature neurons. **D.** Schematic representation of neurogenesis and timeline of marker expression from NSC (left) to mature granule neuron (right).

**Fig 3:**
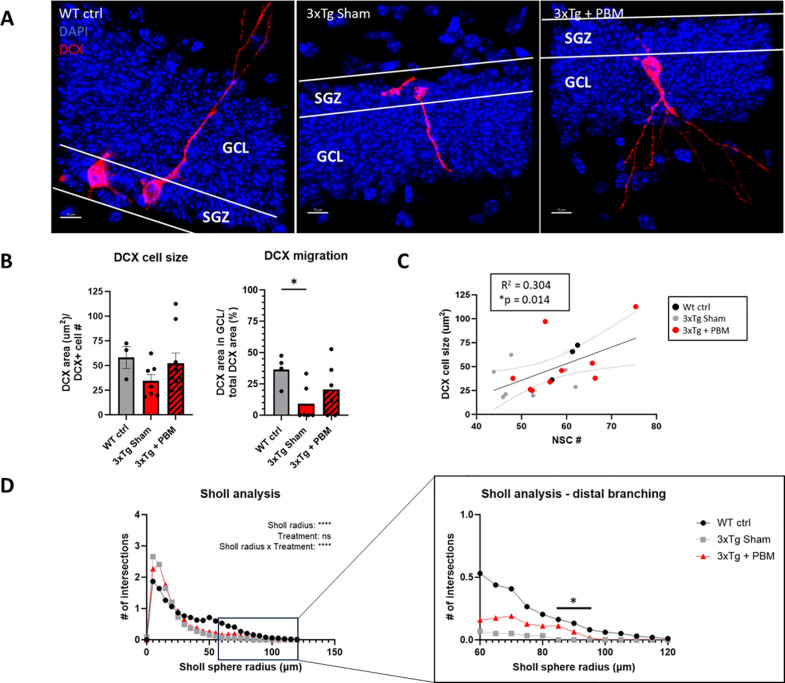
DCX+ cell size, migration, and dendritic arborization are partially rescued by PBM treatment in 3xTg mice. **A.** Representative images of DCX+ newborn neurons. (Scale bars = 10µm) **B.** Quantification of DCX cell size shows no significant differences between groups, but DCX migration into the GCL is significantly reduced in 3xTg Sham, but not 3xTg + PBM compared to WT (One-way ANOVA, *p < 0.05), **C.** Simple linear regression shows a positive correlation between DCX cell size and NSC number. **D**. Sholl concentric sphere analysis of DCX+ cells shows dystrophic sprouting of multiple dendrites in close proximity to the soma in 3xTg mice, and severely diminished dendritic complexity at increasing Sholl sphere radii. *Left:* two-way repeated-measures ANOVA shows significant effect of Sholl radius on the number of intersections and a significant interaction between Sholl radius and treatment, but no overall effect of PBM treatment on number of Sholl intersections. *Right:* Multiple comparisons between groups show significant differences between WT ctrl and 3xTg Sham, but not 3xTg + PBM in the number of intersections between 85 and 95µm Sholl radii.

Adult hippocampal neurogenesis (AHN) is associated with improved memory and synaptic plasticity [14], as well as cognitive resilience to AD neuropathology [16]. Therefore, we examined whether PBM therapy could rescue AHN, which is severely compromised in 3xTg-AD mice by 11 months of age, prior to development of gross Aβ and tau pathology [24]. First, we quantified Sox2+ neural stem cells (NSC) in the dentate gyrus subgranular zone (SGZ). Although Sox2 is not exclusive to NSC in the mammalian brain, and in fact is expressed in astroglia throughout the brain parenchyma, it has been demonstrated that within the SGZ neurogenic niche, Sox2+ cells, even those with astroglial morphology are proliferation competent and capable of generating neurons [25,26]. We found that PBM-treated 3xTg-AD mice had a higher number of NSC per mm (58.94 ± 8.68) than Sham-treated 3xTg AD mice (50.05 ± 7.37), and were nearly restored to WT levels (59.96 ± 2.50) (Fig 1). To quantify neurogenesis we stained for the microtubule-associated protein DCX, which labels transient-amplifying neural progenitors and immature neurons [27], and calretinin (CR), which is expressed in postmitotic newborn neurons prior to maturation at which point Calbindin (CB) expression begins [28]. We found that DCX+ and DCX+/CR+ cells were significantly reduced in the DG of aged 3xTg AD mice as compared to age-matched WT mice. By contrast, we found no differences in the number of CR+ cells between Tg AD mice and wt control (Fig 1A and 1B). We found that PBM did not have any significant effect on the number of DCX+, DCX+/CR+, or DCX-/CR+ cells in the DG of 3xTg AD mice. Notably, we found that, out of the total number of neural progenitors and immature neurons (calculated as the sum of DCX+, DCX+/CR+, and CR+ cells), the percentage of DCX-/CR+ cells was significantly higher in both 3xTg-AD groups (Fig 2C). Proper maturation of DCX+ neuroblasts into functional granule neurons involves migration of the soma out of the SGZ and into the granule cell layer proper (GCL) and projection of an apical dendrite through the GCL and into the molecular layer (ML) where granule neurons synapse with the rest of the hippocampal circuitry [13]. Area analysis of DCX+ cells in the SGZ and the GCL showed no differences in DCX+ cell size, but 3xTg Sham had a significantly reduced fraction of DCX in the GCL compared to WT, but 3xTg + PBM did not (Fig 3B), indicating partial rescue of migration and integration of newborn granule neurons into the GCL. Simple linear regression analysis showed a positive correlation between DCX cell size and NSC # (Fig 3C).

### PBM partially rescues dendritic projection and arborization of newborn neurons in aged 3xTg-AD mice

To further examine the maturation state of DCX+ newborn neurons in our model, we quantified dendrite length and arborization using the Sholl technique. Our analysis found a significant interaction between treatment and Sholl radius on the number of Sholl sphere intersections. Interestingly, the longest recorded Sholl intersection for any 3xTg Sham animal was at 75µm, compared to 100µm for 3xTg + PBM and 120µm for WT (Fig 3D).

### PBM increases extracellular Aβ deposition, microgliosis in the hippocampus of aged 3xTg-AD mice

**Fig 4:**
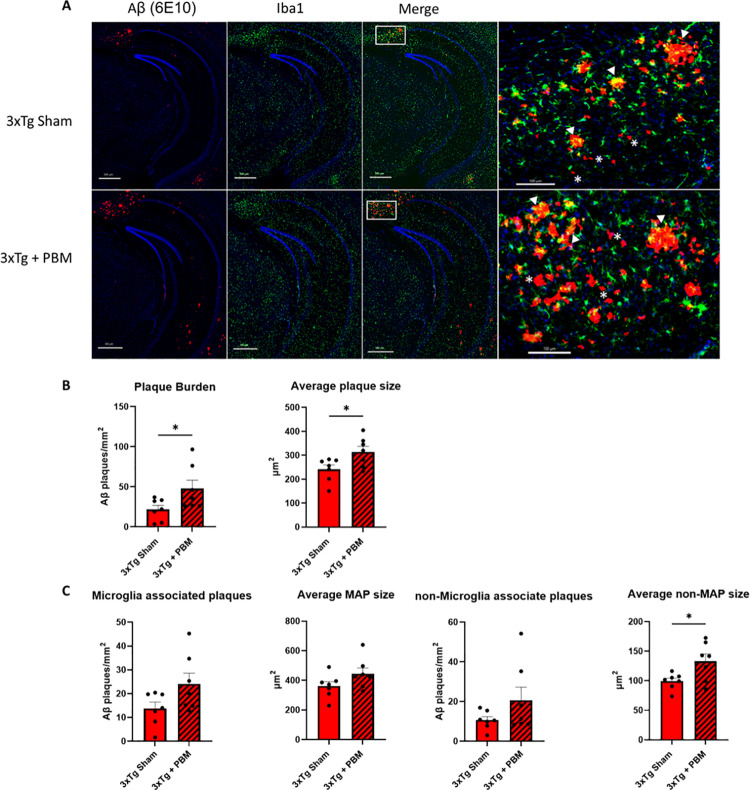
One month of PBM therapy led to increased Aβ plaque deposition, and larger average size of non-microglia associated plaques. **A**. Representative images of whole hippocampal sections showing extracellular Aβ deposits, labeled with 6E10 primary antibody, and microglia labeled with Iba1 (Dapi labeled in blue, scale bar = 500μm). Enlarged panel showing microglia associated plaques (MAP; arrowheads) and non-microglia associated plaques (non-MAP; asterisks) in the subiculum (scale bar = 100μm). **B**. PBM appears to increase the overall deposition of extracellular Aβ plaques and average plaque size. **C.** Quantitative analysis shows no change in the frequency of microglia association with plaques. Microglia-associated plaques (MAP) are on average similar in size between groups, whereas non-MAP are significantly larger in 3xTg + PBM animals. (Unpaired t-test. *p < 0.05).

**Fig 5:**
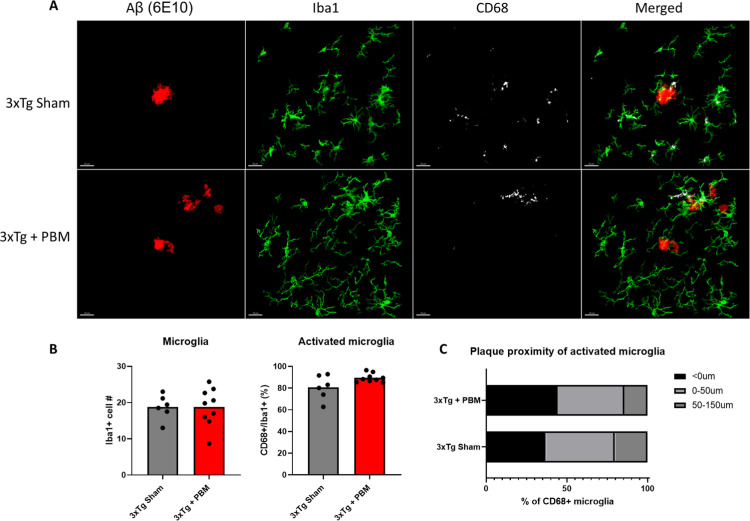
PBM induces focal activation of microglial phagocytosis around Aβ plaques. **A.** Representative confocal images of Aβ plaques (6E10), microglia (Iba1) and CD68 showing diffuse microglial phagocytosis away from plaques in sham compared to localized phagocytosis around plaques in PBM-treated animals. **B.** Quantitative analysis of total and activated microglia show no overall differences in the neuroinflammatory environment surrounding Aβ plaques. (Unpaired t-test). **C.** Analysis of the distribution of CD68+ microglia shows a trend of higher concentration of phagocytic cells in contact with plaques, and decreasing phagocytosis away from plaques. (Two-way ANOVA).

Extracellular accumulation of Aβ peptide into plaques is one of the core neuropathological biomarkers for AD [29]. Due to the observed changes in AHN, we restricted our neuropathological assessment to the hippocampus. In order to study the effect of PBM on Aβ deposition and microgliosis, brain sections were immunostained with an Aβ-specific antibody (6E10) and antibodies against Iba1 and CD68 (markers of total and activated microglia respectively) (Fig 4A). We found that, in our aged 3xTg AD mouse model, four weeks of 670nm PBM increased extracellular Aβ deposition (plaques) in the hippocampus in both number (47.65 ± 28.13 plaques/mm^2^ vs. 21.78 ± 13.48) and average plaque size (314.1 ± 61.73μm^2^ vs. 240.9 ± 48.85) (Fig 4B). To investigate the relationship between Aβ plaque deposition and microgliosis, we examined entire hippocampal sections to assess the association of microglia with plaques. We defined microglia-associated plaques (MAP), as plaques that have microglia touching the plaque (MAP: Iba1 ≤ 0μm from the plaque) and non-MAP as plaques without associated microglia. We found no differences in the number and size of MAPs between Tg Sham and Tg + PBM groups. However, non-MAP in 3xTg + PBM animals were on average significantly larger than non-MAP in 3xTg Sham (133.0 ± 32.22 vs. 99.23 ± 13.69) (Fig 4C). To better understand the inflammatory microenvironment surrounding the plaques, we analyzed Iba1 and the lysosomal marker CD68, to identify total and actively phagocytic microglia respectively, in 60X-magnification confocal microscope images of individual plaques. We found that, although there were no differences between treatment groups in the inflammatory markers analyzed (Fig 5B), PBM altered the distribution of activated microglia such that more CD68+ microglia were more likely to be found in contact with plaques, and less likely 50μm or more away from plaques (Fig 5C).

### AT8+ cell number is unchanged by PBM treatment in aged 3xTg-AD mice

**Fig 6:**
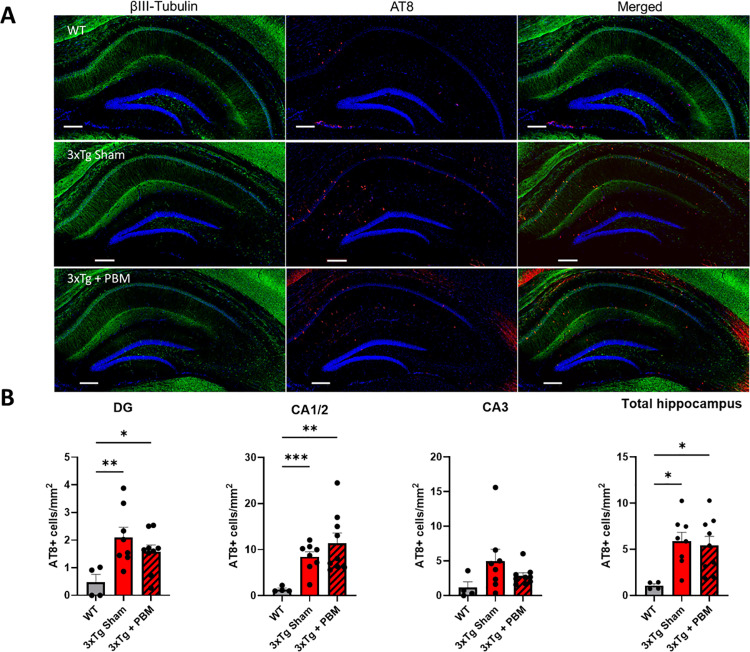
Intracellular tau hyperphosphorylation in the hippocampus is not significantly altered by PBM treatment in aged 3xTg-AD mice. **A.** Representative images of hippocampal sections showing extensive intracellular accumulation of pTau (AT8, red; BIII-tubulin, green; DAPI, blue). (Scale bar = 200µm) **B.** Regional quantifications of AT8+ cells show significant accumulation of intracellular pTau throughout the hippocampus of 3xTg-AD mice with no apparent effect of PBM treatment (One-way ANOVA. *p<0.05, **p<0.01, ***p<0.001).

The second core biomarker for AD diagnosis, tau neurofibrillary tangles (NFT), are the best-known predictor of cognitive decline in AD and related dementias [30–32]. To determine whether tau neuropathologic changes accompanied the cognitive improvement seen with PBM treatment, we investigated hyperphosphorylated tau (pTau) content in the hippocampus using the anti-pTau antibody AT8 (pSer202, pThr205; Invitrogen). Our investigation found significant intracellular pTau deposition in 3xTg mice with occasional AT8+ cells appearing in WT as well. There were no statistically significant differences in the number of AT8+ cells between sham- and PBM-treated 3xTg mice in the DG, CA1/2, or CA3 (Fig 6B).

### Soluble pTau is significantly reduced with PBM treatment

**Fig 7:**
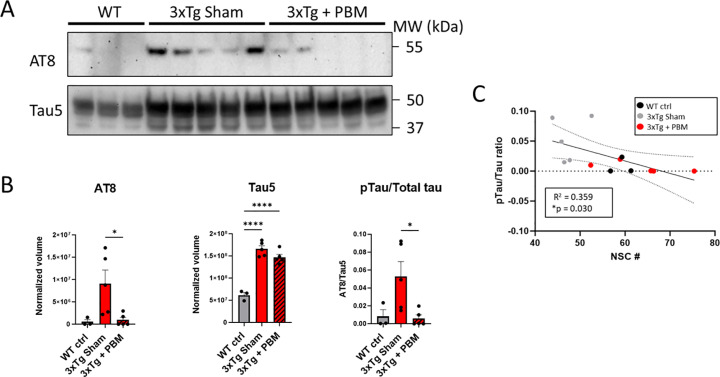
PBM significantly reduces hyperphosphorylation of tau with no effect on total tau expression. **A**. Western blots of total hippocampus detergent-soluble protein extract show decreased hyperphosphorylated tau (AT8: pSer202, pThr205) in 3xTg + PBM compared to Sham with no corresponding decrease in total tau expression. **B**. Quantitative analysis of AT8 and Tau5 western blots normalized to total protein and pTau to total tau ratio. (One-way ANOVA. *p<0.05, ****p<0.0001). **C**. Simple linear regression analysis shows a negative correlation between pTau/Tau ration and neural stem cell number.

Despite the strong correlation of NFT content with cognitive decline, small soluble aggregates, rather than large fibrillar deposits are known to be the primary neurotoxic species of tau [33,34]. Because this PBM treatment protocol has already been shown to reduce oligomeric tau in the 12-month-old 3xTg-AD mouse [35], and our immunofluorescence analysis was incapable of differentiating between soluble oligomeric and insoluble fibrillar pTau, we employed a western blot technique in which we isolated only detergent-soluble hippocampal proteins and quantified pTau and total tau content using AT8 and Tau5, respectively. Our analysis showed significantly reduced AT8 band volume in 3xTg + PBM compared to 3xTg Sham (Fig 7B) and confirmed overexpression of tau in both 3xTg-AD groups compared to WT with no effect of PBM treatment on total tau. Interestingly, we found a significant negative correlation between the ratio of phosphorylated to total tau and NSC# (Fig 7C). These results indicate a specific effect of PBM treatment on the hyperphosphorylation of tau, rather than a global reduction in tau expression.

## Discussion

PBM has been previously demonstrated to induce synaptic resilience to Aβ oligomer toxicity, increase synaptic autophagy, and facilitate reductions in oligomeric tau accumulation in animal models of amyloidosis and tauopathy, and in a middle-aged 3xTg-AD mouse model [17,18]. Here we show that the same PBM protocol in an aged 3xTg-AD mouse model of advanced AD was sufficient to increase NSC pool, partially rescue maturation and integration of newborn neurons in the GCL, and reduce hyperphosphorylated tau in the hippocampus.

### Neurogenesis

Adult hippocampal neurogenesis, the process of generating new neurons from neural stem cells (NSC), is known to contribute to learning and memory and its enhancement has been shown to directly improve pattern recognition [36,37].

In addition to the generation of new neurons, NSC confer neuroprotective effects via secreted exosomes which carry miRNA cargo known to modulate synaptic proteins and rescue Aβ-induced impairment of LTP [15]. Here we found that PBM increased NSC number in 3xTg mice, and rescued the elongation and arborization of apical dendrites of newborn neurons. In younger 3xTg-AD mice between 6- and 12-months of age, NSC have been shown to be reduced as much as 80% compared to age-matched WT controls [24,38]. However, even in WT mice and cognitively healthy humans, neurogenesis and NSC # decline progressively with age [39,40] and are believed to decrease earlier and faster in human AD and animal models eventually stabilizing at significantly reduced levels [13,24]. In this study we found a modest increase in NSC# in PBM-treated 3xTg animals compared to sham, however it was not feasible to discern whether the increased NSC # in 3xTg + PBM animals was caused by an increase in NSC proliferation, or a slowing of NSC depletion. Labeling DNA replication with the thymidine analog 5-bromo-2’-deoxyuridine (BrdU) is a common technique for quantifying adult neurogenesis in younger animals. However, BrdU is toxic and its availability for labeling in the brain is heavily dependent on blood-brain barrier (BBB) integrity [41,42]. Additionally, aberrant cell cycle re-entry is a known phenomenon leading to neurodegeneration in AD and has been found to colocalize near 100% with AT8+ tau pathology in aging 3xTg mice [43]. Considering the extent of hippocampal AT8+ tau pathology in these animals there was a high likelihood of false positive labeling by BrdU, therefore it was not used in this study.

For classifying the maturation of newborn hippocampal neurons we used the markers doublecortin (DCX) and Calretinin (CR). CR is a calcium-binding protein frequently expressed in GABA-ergic interneurons throughout the hippocampus [44,45]. However, a specific subpopulation of CR+ cells at the interface between the GCL and the hilus that do not express GABA but do express immature neuronal markers PSA-NCAM and DCX have been identified as postmitotic newborn neurons [28,46,47]. Here we see the number of CR+ cells in the SGZ unchanged regardless of genotype or treatment, but a significant lack of co-expression with DCX in both 3xTg groups, indicating dysfunction in the maturation process of newborn neurons that warrants further investigation.

### AD neuropathological assessment

In multiple plaque-forming transgenic mouse models, PBM has been shown to decrease Aβ plaque burden and inflammation [48–52]. However, in each case PBM treatment was initiated prior to or at the age of onset of amyloid pathology, indicating that PBM is an effective method for preventing or slowing the development of AD neuropathology and neuroinflammation. In this study, we began treatment at 15 months of age, well after the onset of Aβ pathology and cognitive deficits in female 3xTg mice [22,53–57]. We saw an overall trend of increased extracellular Aβ deposition in the hippocampus, a significant increase in the average size of non-microglia associated Aβ deposits, and no significant changes in microglia-mediated neuroinflammation. There is increasing evidence to support the argument that extracellular accumulation of Aβ into insoluble plaques is neuroprotective. Ballesteros-Álvarez et al. (2023) showed that Aβ plaque burden positively correlated with performance on the Morris water maze test in 12- to 13-month old 3xTg mice, and using PET imaging in human AD subjects [58], Rischel et al. (2023) found that increased Aβ plaque deposition occurred in brain regions with higher metabolic activity [59]. PBM is known to increase mitochondrial metabolism and modulate redox homestasis [49,60,61], both of which also contribute to proliferation and survival of NSC and maturation of adult-born neurons [62–64]. Therefore it is possible that the increased Aβ deposition and increased NSC pool are mechanistically distinct phenomena resulting from PBM therapy that may contribute to improved cognitive function in AD.

We also observed that PBM caused significant reductions in hyperphosphorylated tau, but not total tau production in the hippocampus of 3xTg mice. Hippocampal hyperactivity, a known phenomenon in AD [65–67], depletes the hippocampal NSC pool by inducing cell division and predominantly astrocytic differentiation [68]. This hyperactivity is attributed to the dysfunction of GABAergic interneurons in the hilar region of the DG which are particularly susceptible to hyperphosphorylated tau aggregation [38,65,66]. It has been shown that PBM reduces the activity of ERK [69] and GSK3β [70], two kinases which are directly implicated in the hyperphosphorylation of tau in AD [71]. Given the negative correlation between hippocampal AT8 concentration and NSC number, it is reasonable to conclude that the increased NSC number can be attributed to the decrease in hyperphosphorylated tau, however the possibility that PBM has direct effects on NSC proliferation and differentiation cannot be discounted, in fact it has been shown that PBM alters the expression of miRNA known to regulate proliferation and differentiation of NSC in primary hippocampal stem cells cultured from rats exposed to traumatic brain injury [20]. In the AD brain, Aβ accumulation disrupts mitochondrial redox balance leading to oxidative stress and DNA damage [72]. NSC proliferation and differentiation are metabolically demanding processes which transiently generate reactive oxygen species (ROS) and subsequent oxidative damage under healthy conditions [73], but this oxidative stress could become chronic in the AD brain leading to premature depletion of NSC. The primary mechanism of PBM therapy is photon absorption by cytochrome c oxidase of the mitochondrial electron transport chain leading to increased ATP production [74]. This increase in ATP production can coincide with increased ROS production under conditions of low oxidative stress, or decreased ROS production under high oxidative stress conditions [75]. This balancing of redox states could directly contribute to improved survival and proliferation of NSC, but further investigation in an isolated model of neurogenesis is needed.

While a growing body of evidence suggests remote or extracranial PBM can be effective for a variety of CNS disorders, the underlying mechanisms for this effect are unclear, ranging from modulation of bone-marrow derived mesenchymal stem cells [76], to BBB modulation [77], to microbiome interactions [78]. To narrow the scope of this study, we used the transcranial PBM method and specifically excluded peripheral irradiation to investigate the interaction of red light with the 3xTg-AD mouse brain. It has been previously reported that transcranial irradiation with the 670nm Quantum WARP10 LED device used in this study has ~2.5% transmission across the unshaven C57BL/6 mouse scalp and skull to the cortical surface [49], meaning the combined energy delivered to the cortical surface over the course of treatment was ~1.76J/cm^2^. Despite this limited penetrance, transcranial 670nm light has repeatedly shown therapeutic benefit across a wide range of animal models including multiple AD models [17,35,48,49,79]. This may be attributed to the observation that 670nm light induces maximal cytochrome c oxidase activity and subsequent ATP production compared to the rest of the red – near-infrared spectrum [80]. The mouse is an ideal model for demonstrating therapeutic effects of transcranial PBM due to the relative ease of penetration of light through its thin skull. Shorter wavelengths of light are more prone to scattering and thus do not penetrate as deep as longer wavelengths, limiting the feasibility of 670nm PBM in human patients. Studies of large mammals have shown that 808–810nm near infrared light has the best penetration through thicker skull and scalp tissue [81]. Human clinical trials have shown modest improvement in cognitive testing and executive function with one month [82] and three months [83] of continuous PBM treatment with no adverse effects using 1060–1080nm and 810nm LED light, respectively. Because different wavelengths in the red to near-infrared spectrum affect mitochondrial cytochrome c oxidase similarly [80], our handheld 670nm LED device is a useful tool for understanding the underlying molecular and cellular mechanisms of PBM therapy in neurodegenerative disease.

### Limitations

The main limitation of this study is the use of the 3xTg-AD mouse as a model for Alzheimer’s disease. This model has recently received increased scrutiny due to the use of two mutant transgenes (APP_SWE_, PS1_M146V_) found only in familial AD and a tau_P301L_ mutation found in frontotemporal dementia, a primary tauopathy. Although this model recapitulates the primary proteinopathies and cognitive dysfunction found in AD, it is not an accurate model of the 95% of sporadic AD cases with no known genetic cause, so the findings presented in this study may not be considered generalizeable to the broader population of individuals with AD. Additionally, the translatability of 670nm PBM is the subject of much debate considering the limited penetrance and increased scattering of light by human skin. For this reason many commercially available devices deliver 670nm light in combination with longer wavelengths in the 800–1100nm range to maximize energy delivery.

## Conclusions

In conclusion, our study demonstrates that photobiomodulation (PBM) treatment in an aged 3xTg-AD mouse model of advanced Alzheimer’s disease increases the neural stem cell (NSC) pool, accompanied by a partial rescue in the maturation and integration of newborn neurons in the granule cell layer (GCL). PBM also significantly reduces hyperphosphorylated tau levels in the hippocampus, suggesting that PBM may modulate both neurogenic and neuropathological processes in AD. Additionally, while we observed an increase in extracellular Aβ deposition in PBM-treated animals, this may not directly reflect worsening pathology; rather, it could represent a neuroprotective response induced by PBM or a distinct mechanism related to the increased NSC number. These findings underscore the potential of PBM as a therapeutic approach in AD, though further research is needed to explore its effects on NSC-exosome interactions, tau pathology, and motor networks, as well as to assess its feasibility and efficacy in human clinical trials.

## Supporting information

S1 Fig Characterization of representative plaques for confocal analysis of peri-plaque inflammatory microenvironment. (TIF)

S2 Fig Chemiluminescent images of AT8 and Tau5 western blots. A-B. chemiluminescent image of AT8 bands at ~55kD with molecular weight marker superimposed. C-D. Chemiluminescent image of Tau5 bands with molecular weight marker superimposed. E. Total protein image for band normalization using Bio-RAD stain-free total protein visualization.
(TIF)

S1 Data Raw data. (XLSX)

S1 File Uncropped western blot images. (PDF)
