# Supplementary figures and images for: Photobiomodulation therapy increases neural stem cell pool in aged 3xTg-AD mice
Source: PLoS One. 2025 Apr 22;20(4):e0321668. doi: 10.1371/journal.pone.0321668 (PMC12013953; doi:10.1371/journal.pone.0321668)

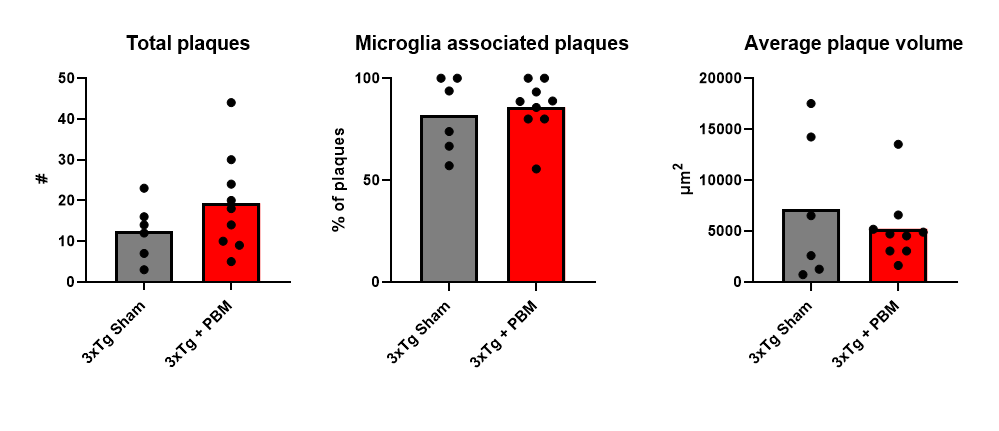

Supplement: S1 Fig — (TIF) [file pone.0321668.s001.tif]

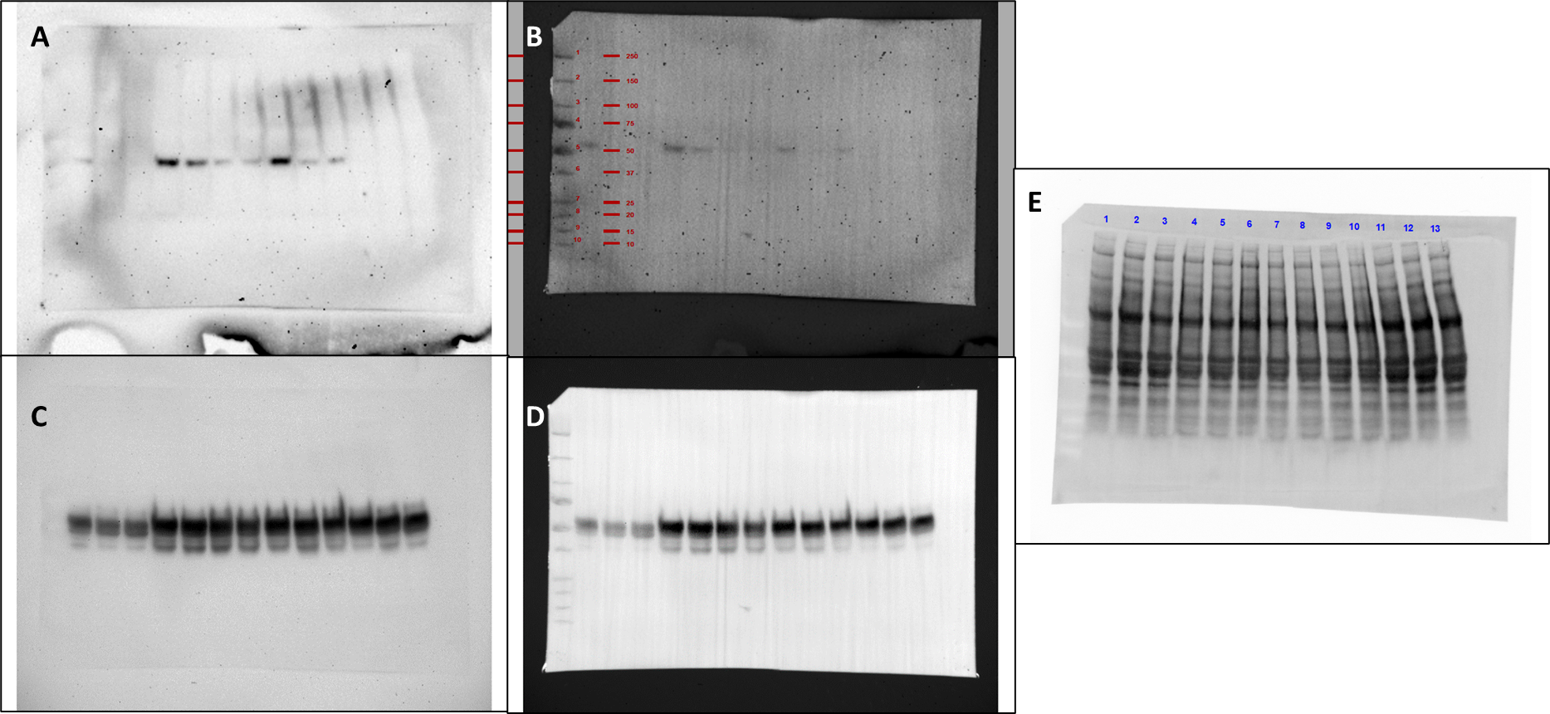

Supplement: S2 Fig — A-B. chemiluminescent image of AT8 bands at ~55kD with molecular weight marker superimposed. C-D. Chemiluminescent image of Tau5 bands with molecular weight marker superimposed. E. Total protein image for band normalization using Bio-RAD stain-free total protein visualization. (TIF) [file pone.0321668.s002.tif]
