# Supplementary material for: Photobiomodulation therapy increases neural stem cell pool in aged 3xTg-AD mice
Source: PLoS One. 2025 Apr 22;20(4):e0321668. doi: 10.1371/journal.pone.0321668 (PMC12013953; doi:10.1371/journal.pone.0321668)

Figure S1 A – raw blot chemiluminescent image of AT8 bands found in figure 7A

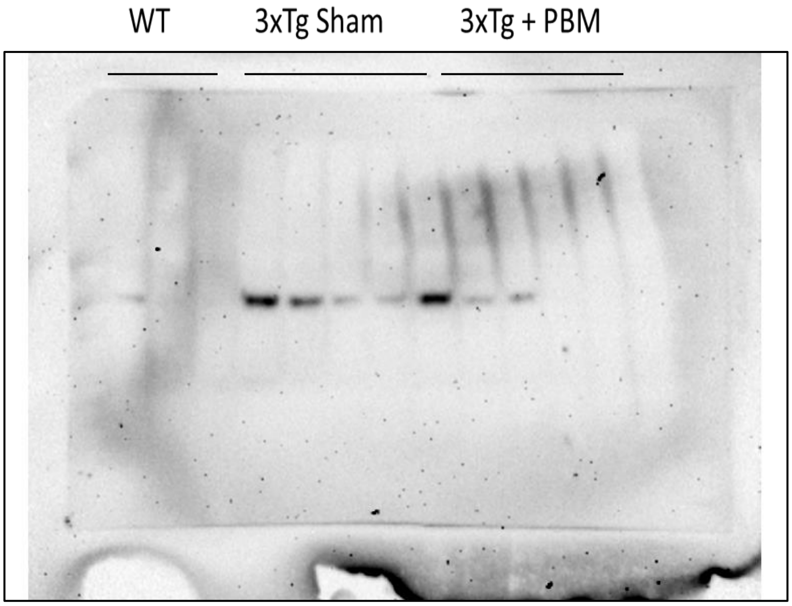

Figure S1 B – raw blot chemiluminescent image above superimposed with colorimetric image of molecular weight ladder showing AT8+ bands at ~55kD

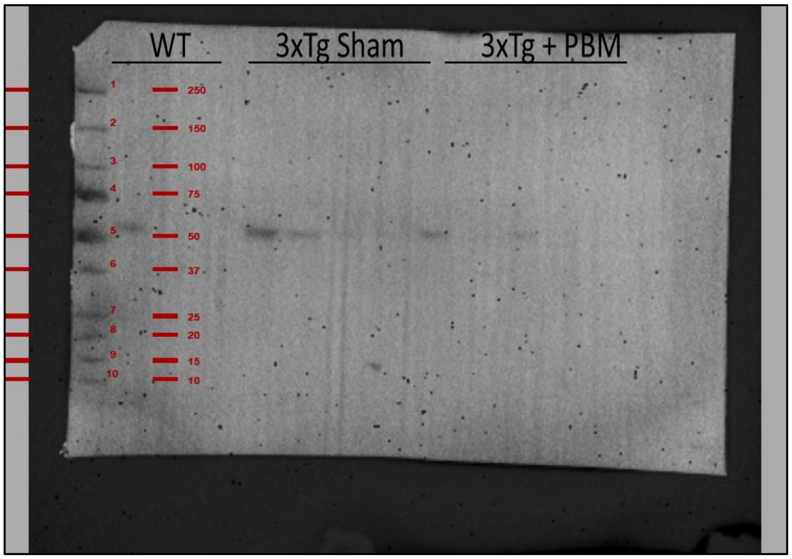

Figure S1 C – Raw chemiluminescent image of Tau5 bands for total tau found in figure 7A

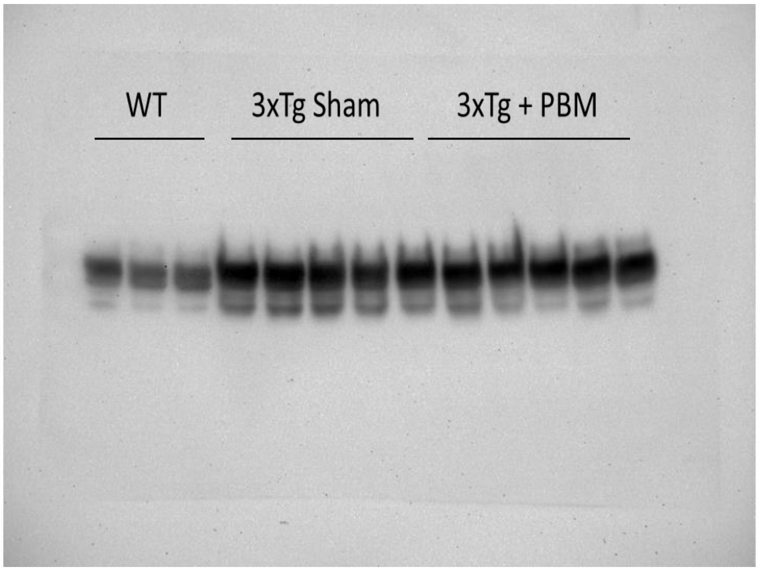

Figure S1 D - raw blot chemiluminescent image above superimposed with colorimetric image of molecular weight ladder showing tau5 bands at 40-55kD

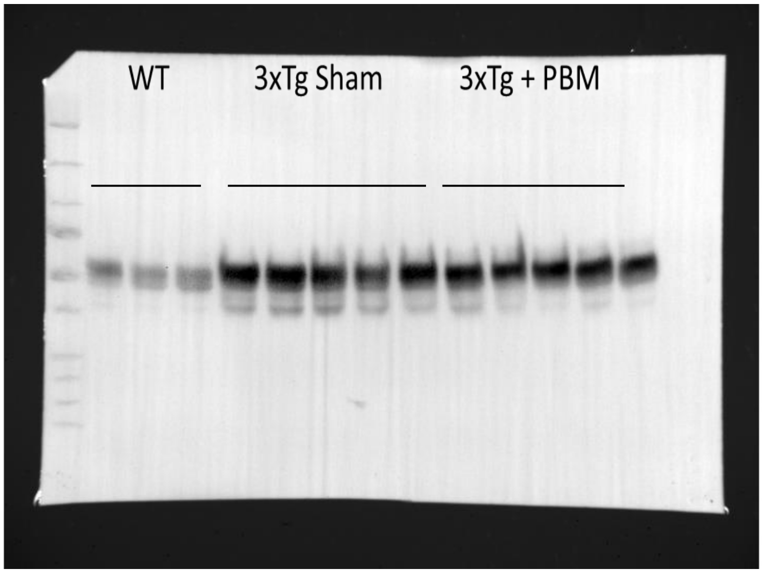

Figure S1 E – UV image of total protein lanes on blot used for normalization of AT8 and Tau5 bands, using Bio-RAD stain-free total protein imaging and ImageLab software

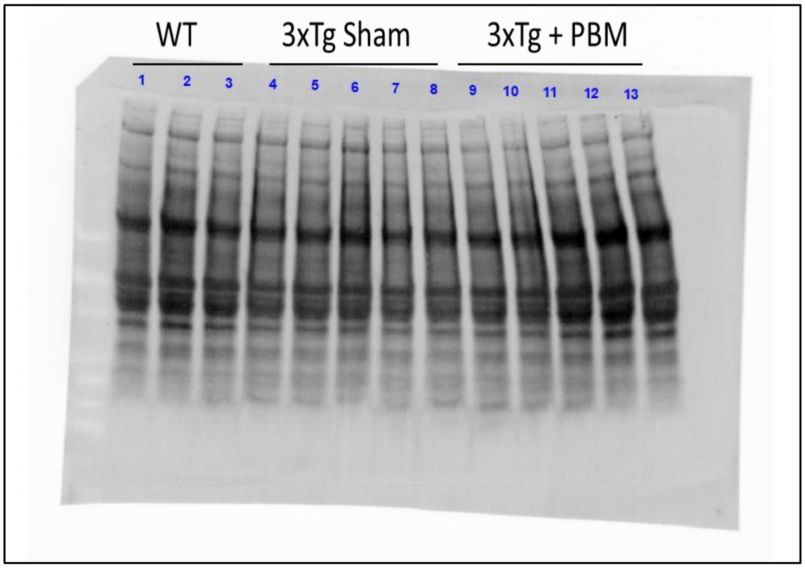

Supplement: S1 File — (PDF) [file pone.0321668.s004.pdf]
